# Microorganisms Causing Community-Acquired Acute Bronchitis: The Role of Bacterial Infection

**DOI:** 10.1371/journal.pone.0165553

**Published:** 2016-10-27

**Authors:** Ji Young Park, Sunghoon Park, Sun Hwa Lee, Myung Goo Lee, Yong Bum Park, Kil Chan Oh, Jae-Myung Lee, Do Il Kim, Ki-Hyun Seo, Kyeong-Cheol Shin, Kwang Ha Yoo, Yongchun Ko, Seung Hun Jang, Ki-Suck Jung, Yong Il Hwang

**Affiliations:** 1 Division of Pulmonary, Allergy and Critical Care Medicine, Hallym University Sacred Heart Hospital, Anyang, Republic of Korea; 2 Seegene Medical Foundation, Seoul, Republic of Korea; 3 Division of Pulmonary, Allergy and Critical Care Medicine, Chuncheon Sacred Heart Hospital, Chuncheon, Republic of Korea; 4 Division of Pulmonary, Allergy and Critical Care Medicine, Kangdong Sacred Heart Hospital, Seoul, Republic of Korea; 5 Myeongmun Clinic of Internal Medicine, Yongin, Republic of Korea; 6 Leejaemyung Clinic of Internal Medicine, Anyang, Republic of Korea; 7 Rapha Clinic of Otolaryngology, Anyang, Republic of Korea; 8 Division of Pulmonary and Critical Care Medicine, Soonchunhyang University Hospital, Cheonan, Republic of Korea; 9 Division of Pulmonary, Allergy and Critical Care Medicine, Yeungnam University Medical Center, Daegu, Republic of Korea; 10 Division of Pulmonary, Allergy and Critical Care Medicine, Konkuk University Hospital, Seoul, Republic of Korea; 11 Division of Pulmonary Medicine, Gwangju Christian Hospital, Gwangju, Republic of Korea; Telethon Institute for Child Health Research, AUSTRALIA

## Abstract

**Background:**

Although acute bronchitis is quite common, there is relatively limited information regarding the microorganisms that are involved in this illness.

**Methods:**

We performed a prospective study of acute bronchitis at 31 hospitals and clinics in Korea from July 2011 to June 2012. Sputum specimens were collected for polymerase chain reaction (PCR) and culture of microorganisms.

**Results:**

Of the 811 enrolled patients, 291 had acceptable sputum specimens that were included for analysis of the etiologic distribution. With multiplex PCR testing, viruses were identified in 36.1% (105/291), most commonly rhinovirus (25.8%) and coronavirus (3.8%). Typical bacteria were isolated in 126/291 (43.3%) patients. Among these patients *Haemophilus influenzae* (n = 39) and *Streptococcus pneumoniae* (n = 30) were isolated most commonly; atypical bacteria were identified in 44 (15.1%) patients. Bacteria-only, virus-only, and mixed infections (bacteria plus virus) accounted for 36.7% (98/291), 17.2% (50/291), and 18.9% (55/291) of infections, respectively. In particular, 52.4% of patients with viral infection had a concurrent bacterial infection, and rhinovirus was the most common virus in mixed infections (40/55). Additionally, infections with typical bacteria were more common in patients with chronic lung disease (*p* = 0.029), and typical bacterial infections showed a trend towards a higher prevalence with older age (*p* = 0.001).

**Conclusions:**

Bacteria were associated with almost half of community-acquired acute bronchitis cases. Additional studies are required to further illuminate the role of bacteria and to identify patient groups most likely to benefit from antibiotic treatment.

## Introduction

Acute bronchitis is an inflammation of the large airways that is characterized by cough and/or sputum that usually lasts one to three weeks [1]. It is one of the most common illnesses among outpatients, and many patients receive antibiotic therapy [1–3].

Traditionally, viruses have been considered the main causative agent of acute bronchitis, possibly explaining the limited benefits of antibiotics [3–5]. However, data regarding the causative microorganisms are still limited. In previous studies, viruses were isolated in 8–23% of community-based cases, not frequently enough to conclude that viruses are the main causal agents for acute bronchitis [6]. Macfarlane et al. identified viruses in only 19% of patients, while typical and atypical bacteria were identified in 25.9% and 23.7% of patients, respectively [7]. In other studies, bacteria were detected in sputum samples in 45% of acute bronchitis patients [8, 9]. In addition, several authors suggested that some patients with acute bronchitis had mixed infections involving both viruses and bacteria. However, the exact prevalence and clinical characteristics of mixed infections have not been well studied [10]. Moreover, it is not clear which subgroup of patients with acute bronchitis could benefit from antibiotic treatments. Recent big data from the UK show that antibiotics substantially reduce the risk of pneumonia after acute bronchitis, particularly in elderly people in whom the risk is highest [11].

Therefore, in the present study, we aimed to investigate the frequencies and characteristics of viral, bacterial, and mixed infections in acute bronchitis in the community. We also hypothesized that the frequencies of these etiologies would vary with underlying lung co-morbidities and age.

## Methods

### Study design

Adult patients with acute bronchitis were prospectively recruited at 31 Korean hospital outpatient departments and primary clinics between July 2011 and June 2012 (6 university-affiliated teaching hospitals, 5 non-teaching community hospitals, and 20 primary clinics). Sputum samples for Gram stains, conventional cultures, and polymerase chain reaction (PCR) were collected from each patient before any medications (including antibiotics) were prescribed. Medications were chosen at the physicians’ discretion. The study protocol was approved by the Institutional Review Board of Hallym University Sacred Heart Hospital (the principal institute, 2011-I049) and each participating hospital. All participants provided informed written consent.

Patients were eligible if they were ≥18 years old and visited the outpatient clinic because of cough (duration < 1 month) with sputum production. Acute bronchitis is a clinical diagnosis, and therefore, a wrong diagnosis is possible. Coughing symptom may have almost all respiratory illnesses as a differential diagnosis. However, symptoms such as sputum production, after carefully discriminating from postnasal drip, could also lead to a diagnosis of lower respiratory inflammation. Patients with typical upper respiratory infection (URI) and symptoms of influenza or influenza-like illness (ILI) during the epidemic period were excluded by participating physicians. We tried to rule out URI by conducting detailed medical interviews, throat examination, and by auscultation. Typically, URI was defined as an infection affecting patients presenting with key symptoms such as sore throat, and nasal symptoms (nasal obstruction, runny nose) with cough. ILI was defined as an abrupt onset of fever with non-productive cough or sore throat [12]. The period of the influenza epidemic was determined by means of a national respiratory virus surveillance system which was broadcast weekly [13]. In some cases, chest radiographs were done at the investigating physician’s discretion, in order to rule out pneumonia. Other exclusion criteria were: 1) history of antibiotic treatment < 7 days before the visit, 2) exacerbation of chronic lung disease within 6 months, 3) active lesion on the chest or paranasal sinus radiographs (when available), 4) immunocompromised status, and 5) confirmed alternative cause for the cough (e.g., drugs [newly started on angiotensin-converting enzyme inhibitors], pneumonia, allergic rhinitis, sinusitis, or gastro-esophageal reflux). Stable chronic lung disease patients were not excluded.

### Data collection

Clinical information was collected by the investigators at outpatient clinics. Data on chronic respiratory diseases, such as asthma, bronchiectasis (BE), and chronic obstructive pulmonary disease (COPD), were collected by reviewing the history and medical records at the enrollment stage of the study. We also investigated the characteristics of the cough (paroxysms, inspiratory whooping, or post-tussive vomiting). At enrollment, sputum specimens were collected from all patients. Sputum amount was classified as scanty (<10 cc/day), moderate (10–50 cc/day), or large (>50 cc/day), and sputum color was classified as white, yellow, brown, or green. The latter three colors were considered to be purulent sputum [14]. Sputum specimens were transferred at room temperature to a central laboratory, and plated on culture media on the day of collection. As this study was based on data obtained from outpatient clinics, we enrolled subjects during working days (5 days a week) [15]. Sputum specimens were considered acceptable if they satisfied Murray-Washington classification degrees IV (10–25 epithelial cells and >25 PMNs per field) or V (<10 epithelial cells and >25 PMNs per field) [16]. Sputum specimens were cultured using routine bacteriological procedures as per guidelines from Clinical and Laboratory Standard Institute [17].

#### Multiplex reverse transcriptase (RT)-PCR for viruses

Multiplex RT-PCR was performed using the DiaPlexC^™^ RV13 Detection Kit (Solgent, Daejeon, South Korea). We used a viral primer panel targeting influenza A and B; respiratory syncytial virus; parainfluenza virus 1, 2, and 3; coronavirus 229E and OC43; human metapneumovirus (hMPV); enterovirus, rhinovirus; human bocavirus; and adenovirus [18]. RT-PCR was performed using a C1000 Touch^™^ Thermal Cycler (Bio-Rad, Hercules, CA, USA).

#### PCR for atypical bacteria

PCR assays for the detection of *Mycoplasma pneumoniae* and *Chlamydophila pneumoniae* were performed as previously described with primers targeting the p1 adhesin gene of *M*. *pneumoniae* and the 16S rRNA gene of *C*. *pneumoniae* [19]. For *Legionella pneumophila*, the primers were targeted at specific regions within the 5S rRNA gene (5'-ACTATAGCGATTTGGAACCA-3' and 5'-GCGATGACCTACTTTCGCAT-3') [20]. PCR testing for *Bordetella pertussis* was performed as described by Nelson et al. [21]. We used the repeated-insertion sequence: primers BP1 (5'-GATTCAATAGGTTGTATGCATGGTT-3') and BP2 (5'-TTCAGGCACACAAACTTGATGGGCG-3'). PCR assays for viruses and atypical bacteria were performed, regardless of sputum acceptability (S1 File).

### Data analysis

Categorical variables were analyzed using the chi-square test or Fisher’s exact test. Continuous variables were analyzed using Student’s *t*-tests or the Mann-Whitney *U*-test. A *p-*value <0.05 was considered statistically significant. All analyses were conducted using SPSS statistical software (IBM SPSS Statistics version 21; IBM, Armonk, NY, USA).

## Results

### Baseline characteristics

We enrolled 811 patients: 526 from primary clinics, 111 from non-teaching community hospitals, and 174 from university-affiliated teaching hospitals. The mean patient age was 48.2 ± 16.8 years and 357 (44.0%) were men (Table 1). Acceptable sputum specimens were received from 291 (35.9%) patients. The mean cough duration before outpatient visits was shorter (acceptable: 6.98 days vs. unacceptable: 8.67 days, p < 0.001), the sputum amount was larger (moderate to large amount; acceptable: 53.6% vs. unacceptable: 34.4%, p < 0.001), and purulent sputum was more common among patients with acceptable sputum samples than in patients with unacceptable sputum samples (acceptable: 56.7% vs. unacceptable: 43.2%, p < 0.001). Lower respiratory signs, such as wheezing, crackles, rhonchi, and stridor, were more common in the acceptable sputum group (S1 Table).

**Table 1:** Clinical characteristics of 811 patients with acute bronchitis.

Variables	Total (n = 811)	Acceptable sputum (n = 291)*
Mean age, yr	48.2 ± 16.8	49.4 ± 17.3
Gender, male	357 (44.0)	123 (42.3)
Current smoker	165 (20.3)	56 (19.2)
Systemic disease		
Hypertension	127 (15.7)	44 (15.1)
Diabetes mellitus	49 (6.0)	21 (7.2)
Chronic heart disease	17 (2.1)	4 (1.4)
Chronic kidney disease	4 (0.5)	1 (0.3)
Cerebrovascular disease	5 (0.6)	2 (0.7)
Pulmonary comorbidities	152 (18.7)	76 (26.1)
Asthma only	66 (8.1)	29 (10.0)
COPD only	41 (5.1)	27 (9.3)
Bronchiectasis only	31 (3.8)	12 (4.1)
Combined lung diseases	14 (1.7)	8 (2.7)
Symptoms		
Duration of cough, day	8.06 ± 6.49	6.98 ± 5.15
Sputum amount (≥moderate)	335 (41.3)	156 (53.6)
Purulent sputum	389 (48.0)	165 (56.7)
Rhinorrhea	283 (34.9)	111 (38.1)
Sore throat	272 (33.5)	108 (37.1)
Dyspnea	89 (11.0)	33 (11.3)
Fever	32 (3.9)	16 (5.5)
Chest pain	94 (11.6)	32 (11.0)
Signs		
Wheezing	64 (7.9)	34 (11.7)
Crackle	74 (9.1)	35 (12.0)
Rhonchi	240 (29.6)	108 (37.1)
Stridor	16 (2.0)	10 (3.4)

* Acceptable sputum was defined by satisfying Murray-Washington classification degree IV or V.

Abbreviations: COPD, chronic obstructive pulmonary disease. Combined lung diseases include Asthma + COPD, asthma + bronchiectasis, COPD + bronchiectasis, and all three diseases together.

### Microbiological findings

**Fig 1:**
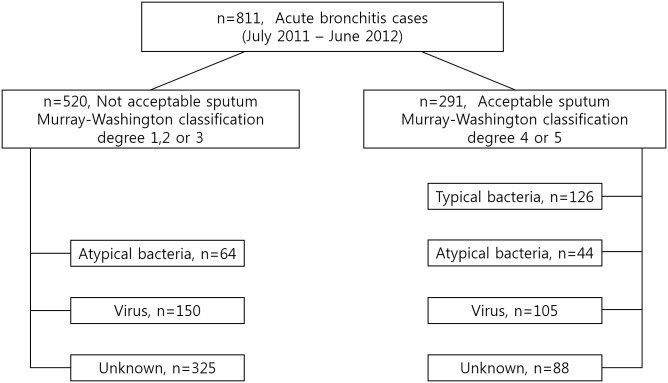
Etiology of acute bronchitis in 291 patients with acceptable sputum and 520 patients with unacceptable sputum.

**Fig 2:**
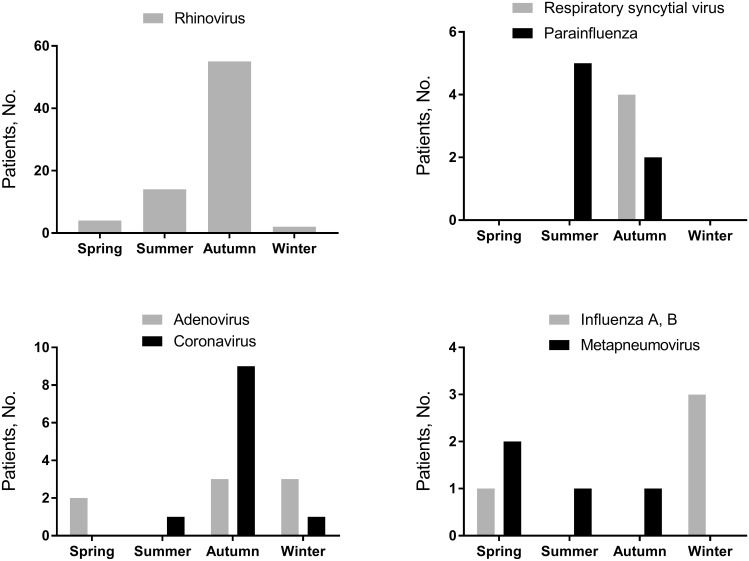
Seasonal distribution of the causative viruses identified in 291 patients with acute bronchitis.

Of the 811 enrolled patients, 291 (35.9%) had acceptable sputum specimens that were included for analysis of the etiologic distribution. With multiplex PCR testing, viruses were identified in 36.1% (105/291) (Fig 1), most commonly rhinovirus (25.8%) and coronavirus (3.8%). Co-infection with >1 virus was detected in 16 (5.5%) patients, most commonly rhinovirus plus enterovirus (6/291) and rhinovirus plus adenovirus (4/291). Viruses were identified significantly more during the fall and winter (42.5% September–February vs. 26.5% March–August, p = 0.005). Rhinovirus showed a peak incidence during the fall (Fig 2). PCR tests for atypical pathogens were positive in 15.1% (44/291) of patients (M. pneumoniae, n = 18; C. pneumoniae, n = 1; L. pneumophila, n = 15; and B. pertussis, n = 11). Overall, 165 strains of typical bacteria were cultured from 126 (43.3%) patients. *Haemophilus influenzae* (13.4%, 39/291) and *Streptococcus pneumoniae* (10.3%, 30/291) were isolated most frequently. Co-infection with >1 typical bacteria was found in 6.2% (18/291) of investigated patients (Table 2).

**Table 2:** Distribution of the microorganisms identified in 291 patients with acute bronchitis with acceptable sputum.

Etiologic agents (isolated patients)	No. (%)
Viral pathogens (n = 105/291)	
Rhinovirus	75 (25.8)
Coronavirus	11 (3.8)
Adenovirus	8 (2.7)
Parainfluenza virus	7 (2.4)
Enterovirus	6 (2.1)
Human metapneumovirus	4 (1.4)
Respiratory syncytial virus	4 (1.4)
Influenza A	3 (1.0)
Human bocavirus	2 (0.7)
Influenza B	1 (0.3)
Multiple viral co-infection	16 (5.5)
Atypical bacterial pathogens (n = 44/291)	
*Mycoplasma pneumoniae*	18 (6.2)
*Legionella pneumophila*	15 (5.2)
*Bordetella pertussis*	11 (3.8)
*Chlamydophila pneumoniae*	1 (0.3)
Typical bacterial pathogens (n = 126/291)	
*Haemophilus Influenzae*	39 (13.4)
*Streptococcus pneumoniae*	30 (10.3)
*Klebsiella pneumoniae*	20 (6.9)
*Moraxella catarrhalis*	17 (5.8)
*Staphylococcus aureus*	14 (4.8)
*Pseudomonas aeruginosa*	8 (2.7)
*Haemophilus parainfluenzae*	5 (1.7)
Group B *β-hemolytic Streptococcus*	3 (1.0)
Group A *β-hemolytic Streptococcus*	2 (0.7)
*Stenotrophomonas maltophilia*	2 (0.7)
*Streptococcus agalactiae*	1 (0.3)
Other Gram negative rod	5 (1.7)
Multiple typical bacterial co-infection	18 (6.2)

Mixed infection with at least one bacterium (typical or atypical) and at least one virus was demonstrated in 18.9% (55/291) of patients with acceptable sputum. Bacteria-only and virus-only infection were observed in 33.7% (98/291) and 17.2% (50/291) of the patients, respectively, while no organism was identified in the remaining 30.2% (88/291) of patients (Fig 1). More than 50% of patients with viral infection (55/105) had a concurrent infection with at least one bacterial agent. Rhinovirus was the most common virus in mixed infections with bacteria (72.7%, 40/55). The distribution of mixed infections in patients with acceptable sputum is shown in S2 Table.

### Microorganisms in patients with and without underlying lung disease

The overall prevalence of viral infections was similar for patients with and without chronic lung disease (Table 3). Infections caused by atypical pathogens, especially *M*. *pneumoniae* were observed less frequently in chronic lung disease patients, whereas infections with typical bacteria were detected more commonly in patients with chronic lung disease (53.9% vs. 39.5% *p* = 0.029). However, the incidences of co-infection (i.e., by multiple bacterial agents) and mixed infection (i.e., with both bacterium and virus) did not differ significantly between patients with and without chronic lung disease.

**Table 3:** Distribution of the microorganisms identified in 291 patients with acute bronchitis according to lung co-morbidities.

Microorganism	Without chronic lung disease (n = 215)	Chronic lung disease, Total (n = 76)	Asthma (n = 29)	COPD (n = 27)	BE (n = 12)	Combined lung diseases(n = 8)
Virus	75 (34.9)	30 (39.5)	13 (44.8)	11 (40.7)	3 (25.0)	3 (37.5)
Rhinovirus	54 (25.1)	21 (27.6)	11 (37.9)	6 (22.2)	2 (16.7)	2 (25.0)
Coronavirus	9 (4.2)	2 (2.6)	2 (6.9)	0 (0)	0 (0)	0 (0)
Adenovirus	8 (3.7)	0 (0.0)	0 (0)	0 (0)	0 (0)	0 (0)
Parainfluenza virus	2 (0.9)	5 (6.6)*	1 (3.4)	2 (7.4)	1 (8.3)	1 (12.5)
Enterovirus	4 (1.9)	2 (2.6)	2 (6.9)	0 (0)	0 (0)	0 (0)
Influenza A	2 (0.9)	1 (1.3)	0 (0)	1 (3.7)	0 (0)	0 (0)
Respiratory syncytial virus	2 (0.9)	2 (2.6)	0 (0)	2 (7.4)	0 (0)	0 (0)
Atypical bacteria	37 (17.2)	7 (9.2)	1 (3.4)	3 (11.1)	2 (16.7)	1 (12.5)
*M*. *pneumoniae*	17 (7.9)	1 (1.3)*	0 (0)	0 (0)	1 (8.3)	0 (0)
*L*. *pneumophila*	8 (3.7)	7 (9.2)	1 (3.4)	3 (11.1)	2 (16.7)	1 (12.5)
*C*. *pneumoniae*	1 (0.5)	0 (0.0)	0 (0)	0 (0)	0 (0)	0 (0)
*B*. *Pertussis*	11 (5.1)	0 (0.0)	0 (0)	0 (0)	0 (0)	0 (0)
Typical bacteria	85 (39.5)	41 (53.9)*	12 (41.4)	16 (59.3)*	7 (58.3)	6 (75.0)
*H*. *Influenzae*	27 (12.6)	12 (15.8)	2 (6.9)	5 (18.5)	2 (16.7)	3 (37.5)
*S*. *pneumoniae*	20 (9.3)	10 (13.2)	5 (17.2)	2 (7.4)	0 (0)	3 (37.5)*
*K*. *pneumoniae*	13 (6.0)	7 (9.2)	4 (13.8)	2 (7.4)	1 (8.3)	0 (0)
*M*. *catarrhalis*	11 (5.1)	6 (7.9)	2 (6.9)	3 (11.1)	0 (0)	1 (12.5)
*S*. *aureus*	11 (5.1)	3 (3.9)	1 (3.4)	0 (0)	2 (16.7)	0 (0)
*P*. *aeruginosa*	4/ (1.9)	4 (5.3)	0 (0)	2 (7.4)	1 (8.3)	1 (12.5)
Others	11 (5.1)	7 (9.2)	2 (6.9)	2 (7.4)	3 (25.0)*	0 (0)
Co-infection with multi-bacteria	11 (5.1)	7 (9.2)	3 (10.3)	0 (0)	2 (16.7)	2 (25.0)
Mixed infection with bacteria and virus	38 (17.7)	17 (22.4)	6 (20.7)	6 (22.2)	2 (16.7)	3 (37.5)

*p < 0.05 for the comparison with patients without underlying chronic lung disease.

Abbreviations: COPD, chronic obstructive pulmonary disease; BE, bronchiectasis. Combined lung diseases include Asthma + COPD, asthma + BE, COPD + BE, and all three diseases together.

### Microorganisms according to age

For viruses, the overall prevalence was higher in younger patients (Table 4, *p* = 0.046). Rhinovirus was the most prevalent cause of infection in <40-year-old patients (34.4%) and showed a trend of declining prevalence with older age (*p* = 0.044). For typical bacterial agents, there was a trend of higher prevalence at older ages (*p* = 0.001). There were no significant differences for atypical pathogen. The prevalence of co-infection and mixed infection did not differ across the three age groups.

**Table 4:** Distribution of the causative microorganisms identified in 291 patients with acute bronchitis according to age group.

	Age 18–39 (n = 96)	Age 40–59 (n = 95)	Age 60- (n = 100)	P value
Virus	42 (43.8)	36 (37.9)	27 (27.0)	0.046
Rhinovirus	33 (34.4)	23 (24.2)	19 (19.0)	0.044
Adenovirus	8 (8.3)	0 (0)	0 (0)	0.001
Parainfluenza virus	0 (0)	3 (3.2)	4 (4.0)	0.159
Coronavirus	5 (5.2)	4 (4.2)	2 (2.0)	0.482
Enterovirus	3 (3.1)	1 (1.1)	2 (2.0)	0.601
Respiratory syncytial virus	1 (1.0)	3 (3.2)	0 (0)	0.157
Influenza A	1 (1.0)	1 (1.1)	1 (1.0)	0.999
Human metapneumovirus	1 (1.0)	3 (3.2)	0 (0)	0.157
Co-infection with multi-virus	12 (12.5)	2 (2.1)	2 (2.0)	0.001
Atypical bacteria	18 (18.8)	14 (14.7)	12 (12.0)	0.416
*M*. *pneumoniae*	8 (8.3)	8 (8.4)	2 (1.0)	0.100
*C*. *pneumoniae*	1 (1.0)	0 (0)	0 (0)	0.361
*L*. *pneumophila*	4 (4.2)	2 (2.1)	9 (9.0)	0.081
*B*. *pertussis*	5 (5.2)	4 (4.2)	2 (2.0)	0.482
Typical Bacteria	34 (35.4)	34 (35.8)	58 (58.0)	0.001
*H*. *Influenzae*	13 (13.5)	9 (9.5)	17 (17.0)	0.468
*S*. *pneumoniae*	7 (7.3)	8 (8.4)	15 (15.0)	0.075
*K*. *pneumoniae*	4 (4.2)	5 (5.3)	11 (11.0)	0.058
*M*. *catarrhalis*	6 (6.3)	5 (5.3)	6 (6.0)	0.943
*S*. *aureus*	4 (4.2)	6 (6.3)	4 (4.0)	0.949
*P*. *aeruginosa*	1 (1.0)	1 (1.1)	6 (6.0)	0.033
Other bacteria	5 (5.2)	5 (5.3)	8 (8.0)	0.415
Co-infection with multi-bacteria	5 (5.2)	5 (5.3)	8 (8.0)	0.415
Mixed infection with bacteria and virus	19 (19.8)	19 (20.0)	17 (17.0)	0.816

### Clinical characteristics according to microorganism group

We compared clinical characteristics according to microorganism groups (Table 5). The bacteria-only group was significantly older (mean age, 52.2 vs. 43.3 years; *p* = 0.004) and included significantly more men (52.0% vs. 34.0%; *p* = 0.037) than the virus-only group. Only sore throat was more common in the virus-only group than in the bacteria-only group (32.7% vs. 52.0%; *p* = 0.023). Patients with mixed infections (55/291) showed intermediate values between the bacteria-only and virus-only groups in terms of mean age, sex ratio, and sore throat prevalence.

**Table 5:** Characteristics of patients with acute bronchitis according to microbiological categories in 291 patients with acceptable sputum (Murray-Washington classification degree IV or V).

	Mixed* (n = 55)	Bacteria only (n = 98)	Virus only (n = 50)	Unknown (n = 88)	*P*-vale Bacteria only vs. Virus only
Mean age	49.0 ± 17.7	52.2 ± 17.6	43.3 ± 17.6	49.9 ±16.1	0.004
Sex, male	23 (41.8)	51 (52.0)	17 (34.0)	32 (36.4)	0.037
Underlying lung disease	17 (30.9)	25 (25.5)	13 (26.0)	21 (23.9)	0.949
Symptoms					
Duration of cough, day	6.5 ± 4.1	6.6 ± 4.4	5.9 ± 5.5	8.3 ± 6.1	0.408
Cough					
Paroxysmal cough	31 (56.4)	49 (50.0)	29 (58.0)	42 (47.7)	0.357
Whooping cough	5 (9.1)	12 (12.2)	7 (14.0)	9 (10.2)	0.763
Post-cough vomit	6 (10.9)	2 (2.0)	2 (4.0)	2 (2.3)	0.487
Sputum color					
White	21 (38.2)	40 (40.8)	28 (56.0)	37 (42.0)	
Purulent	34 (61.8)	58 (59.2)	22 (44.0)	51 (58.0)	0.080
Yellow	32 (58.2)	54 (55.1)	22 (44.0)	44 (50.0)	
Brown	1 (1.8)	1 (1.0)	0 (0.0)	5 (5.7)	
Green	1 (1.8)	3 (3.1)	0 (0.0)	2 (2.3)	
Sputum amount					0.639
Scanty	28 (50.9)	44 (44.9)	22 (44.0)	41 (46.6)	
Moderate	27 (49.1)	49 (50.0)	27 (54.0)	44 (50.0)	
Large amount	0 (0.0)	5 (5.1)	1 (2.0)	3 (3.4)	
Rhinorrhea	27 (49.1)	35 (35.7)	23 (46.0)	26 (29.5)	0.225
Sore throat	26 (47.3)	32 (32.7)	26 (52.0)	24 (27.3)	0.023
Dyspnea	6 (10.9)	14 (14.3)	5 (10.0)	8 (9.1)	0.461
Hemoptysis	2 (3.6)	3 (3.1)	0 (0.0)	3 (3.4)	0.211
Headache	12 (21.8)	21 (21.4)	11 (22.0)	17 (19.3)	0.936
Fever	3 (5.5)	4 (4.1)	6 (12.0)	3 (3.4)	0.069
Chillness	7 (12.7)	8 (8.2)	2 (4.0)	7 (8.0)	0.340
Myalgia	14 (25.5)	24 (24.5)	7 (14.0)	10 (11.4)	0.138
Malaise	9 (16.4)	13 (13.3)	5 (10.0)	8 (9.1)	0.565
Chest pain	3 (5.5)	11 (11.2)	7 (14.0)	11 (12.5)	0.625
Signs					
Wheezing	3 (5.5)	14 (14.3)	5 (10.0)	12 (13.6)	0.461
Crackle	3 (5.5)	12 (12.2)	6 (12.0)	14 (15.9)	0.966
Rhonchi	18 (32.7)	41 (41.8)	16 (32.0)	33 (37.5)	0.245
Stridor	2 (3.6)	4 (4.1)	2 (4.0)	2 (2.3)	1.000

*Mixed infection with at least one bacterium (typical or atypical) and at least one virus.

## Discussion

To the best of our knowledge, this is among the largest prospective multicenter studies regarding the microbiology of acute bronchitis. Notably, about 50% of patients with acute bronchitis and acceptable sputum had evidence of bacterial infection (typical or atypical), a higher frequency than that of viral infection. Also, the distributions of infectious etiologies differed by age and the presence of underlying chronic lung disease. Finally, mixed infections were common, and >50% of patients with viral infections also had bacterial infections.

In recent placebo-controlled randomized trials, antibiotics appeared to provide minimal benefit in treating acute bronchitis [4, 5, 22]. Therefore, it is often assumed that acute bronchitis is primarily a viral disease [4, 5]. Accordingly, previous studies have usually focused on viral etiologies and did not include sputum cultures, or used only serological tests for bacteria, and may have underestimated the bacteria’s role in acute bronchitis [23–25]. Creer et al. detected bacteria and viruses in 26% and 63% of acute bronchitis patients, respectively, prompting the authors to consider bacterial infection relatively uncommon [26]. However, they collected sputum specimens for bacterial cultures from only a portion of patients, most patients submitted swabs and nasal aspirates for viral testing, and sputum specimen adequacy was not discussed. In contrast, we collected sputum specimens from all patients for viral, typical bacterial, and atypical bacterial infection simultaneously.

Although previous studies showed that antibiotics have little benefit in treating acute bronchitis, several factors may have affected the results [4, 5, 27]. First, patients included in such studies were inhomogeneous, and a considerable proportion of them may have had only upper respiratory tract infections (URIs). It is more appropriate to evaluate antibiotic effects in a fully differentiated group of bacterial etiologies. Second, the use of the term ‘bacterial infection’ in the lower respiratory tract does not necessarily imply that there is a requirement for antibiotics. Many of these infections can be cured without antibiotics [28]. Third, even if bacterial infections were treated using antibiotics, subjective symptoms, such as post-infectious cough or upper-airway cough syndrome, might persist [29]. Interestingly, in a randomized controlled trial of patients with URIs, antibiotics were clinically beneficial for a subgroup whose nasopharyngeal secretions contained respiratory bacteria [30]. Although the presence of bacterial agents does not always indicate a disease, antibiotic treatments might be beneficial in subgroups of patients with bacterial etiologies. C-reactive protein or procalcitonin levels may eventually provide an objective marker for evaluating the need for antibiotic treatment [31].

Recent studies have used the term “lower respiratory tract infection (LRTI)” for conditions approximating acute bronchitis. LRTI is characterized by acute cough, with at least one other lower respiratory tract symptom, including purulent sputum, dyspnea, wheezing, chest discomfort, or chest pain [1, 4, 7, 10]. In our study, we attempted to limit patient enrollment to patients with acute bronchitis; however, some URI patients might have been enrolled because of symptom overlap. This potential bias should be considered when interpreting our results [25]. However, the cohort of 291 patients with acceptable sputum could be considered as a purer group of acute bronchitis patients. Patients with acceptable sputum had more sputum production and auscultatory abnormalities, strongly supporting the diagnosis of LRTI [10].

In earlier studies, viral prevalence was 9.2–61.3% and rhinovirus or influenza were most commonly detected [7, 8, 26, 32, 33]. Importantly, not all studies spanned an entire year, and some were limited to the winter influenza season [25, 33]. We enrolled patients for an entire year, but fewer were enrolled during the winter, likely because we excluded those with typical symptoms of influenza during the winter (December–February).

In our study, the most common bacterial agent was *H*. *influenzae*, followed by *S*. *pneumoniae*. Some previous studies found *S*. *pneumoniae* to be the most frequent pathogen [7, 26], while others found *H*. *influenzae* more frequently [28, 34]. However, with the detection of pneumococcal antigens in sputum or urine, or PCR on airway secretions, *S*. *pneumoniae* is found in 17–19% of LRTI cases [7, 26]. Because we only used sputum cultures to detect typical bacteria, the incidence of *S*. *pneumoniae* might have been underestimated. Interestingly, *S*. *pneumoniae* was isolated in older patients twice as commonly than in younger patients (≥60 vs. <40 years: 15.0% vs. 7.3%, *p* = 0.075). Therefore, pneumococcal vaccines may be beneficial in preventing acute bronchitis associated with *S*. *pneumoniae* in older patients. Additional studies are warranted.

Mixed infection occurred in 18.9% of our patients, in 22–32% of patients in previous studies of LRTI [7, 8, 26], and in 6–26% of non-immunocompromised adults with community-acquired pneumonia (CAP) [10]. The most typical combination has been viral-bacterial mixed infection [10, 35]. In our study, rhinovirus was the most common virus in mixed infections. Several studies have suggested that rhinovirus can be pathogenic for LRTI, but it is unclear whether rhinovirus triggers secondary bacterial infection [36–38]. Also, viral-bacterial mixed infections have induced more severe inflammation and disease than individual infections in CAP cases [35, 39, 40]. Clinical features and outcomes have not been studied in patients with mixed infection acute bronchitis or LRTI without pneumonia, and we found no distinct characteristics of mixed infections.

We did not exclude patients with chronic lung disease, a large proportion of the patients evaluated for cough in our clinics. In a recent review, Mohan et al. detected viruses via PCR and RT-PCR in 34.1% of patients with an acute exacerbation of COPD [41]. The same rate (34.1%) was observed for COPD patients in our study. However, none of our COPD or asthma patients were positive for *M*. *pneumoniae*, *C*. *pneumoniae*, or *B*. *pertussis*. Despite potential methodological problems, studies using PCR also found no COPD exacerbations associated with *M*. *pneumoniae* or *C*. *pneumoniae* [42, 43]. In a previous multicenter study, we demonstrated the absence of *B*. *pertussis* in patients with chronic lung disease [44].

We found that the prevalences of rhinovirus, adenovirus, and *M*. *pneumoniae* with acute bronchitis were higher in young adults. This observation is consistent with prior LRTI studies [33]. Conversely, the frequency of typical bacteria was higher in the older age group, as demonstrated in studies of CAP [45, 46]. This result suggests antibiotics may be more beneficial in older patients with acute bronchitis. Petersen et al. reported that antibiotics substantially reduced pneumonia risk after chest infection (acute bronchitis), particularly in elderly patients [11].

In summary, bacterial infections were identified as the etiology for about half of the 35.9% of acute bronchitis patients who had acceptable sputum. The infectious etiologies differed by age and the presence of underlying chronic lung disease. Further, mixed infection with both bacteria and viruses were common. Future research should be directed at the identification of patient groups most likely to benefit from antibiotic treatment.

## Supporting Information

S1 File Supplementary methods. (DOCX)

S1 Table Comparison of clinical characteristics in patients with acceptable sputum and unacceptable sputum. (DOCX)

S2 Table The distribution of mixed infections in patients with acceptable sputum. (DOCX)

## Abbreviations

COPD: chronic obstructive pulmonary disease
PCR: polymerase chain reaction
URI: upper respiratory infection
PMN: polymorpho-neutrophils
LRTI: lower respiratory tract infection
CAP: community-acquired pneumonia
