# Supplementary material for: Microorganisms Causing Community-Acquired Acute Bronchitis: The Role of Bacterial Infection
Source: PLoS One. 2016 Oct 27;11(10):e0165553. doi: 10.1371/journal.pone.0165553 (PMC5082923; doi:10.1371/journal.pone.0165553)
Supplement: S1 File — (DOCX) [file pone.0165553.s001.docx]

**SUPPLEMENTARY INFORMATION**

**Microorganisms causing community-acquired acute bronchitis: the role of bacterial infection**

Ji Young Park, Sunghoon Park, Sun Hwa Lee, Myung Goo Lee, Yong Bum Park, Kil Chan Oh, Jae-Myung Lee, Do Il Kim, Ki-Hyun Seo, Kyeong-Cheol Shin, Kwang Ha Yoo, Yongchun Ko, Seung Hun Jang, Ki-Suck Jung , and Yong Il Hwang

**S1 File. Supplementary methods**

**Nucleotide extraction method**

**DNA extraction**

DNA was extracted from each sputum specimen using the Puregene DNA Isolation Kit (Gentra Systems, Minneapolis, MN, USA). Concentrated sputum specimens were mixed in cell lysis solution and heated at 65°C for 15 min. RNase solution was added and, after protein precipitation, the supernatant was mixed with 300 µl isopropanol and incubated for 5 min at room temperature. After supernatant removal, 70% ethanol was added and centrifuged. The DNA pellet was air dried and added to 100 µl DNA hydration solution [1].

**RNA extraction**

Viral RNA was extracted from samples using the QIAamp® Viral RNA Mini Kit (Qiagen, Hilden, Germany). The sample was first lysed using a lysing buffer with carrier RNA, which enhances the binding of viral nucleic acids to the silica membrane and reduces chance of viral RNA degradation. The mixture was applied to a spin column for removal of contaminants with washing buffers. The extracted RNA was eluted in RNase-free water containing 0.04% sodium azide [2].

**References**

[1] Catten MD, Murr AH, Goldstein JA, Mhatre AN, Lalwani AK. Detection of fungi in the nasal mucosa using polymerase chain reaction. *Laryngoscope*. 2001; **111**: 399-403.

[2] Yam WC, Chan KH, Poon LL, Guan Y, Yuen KY, Seto WH, Peiris JS. Evaluation of reverse transcription-PCR assays for rapid diagnosis of severe acute respiratory syndrome associated with a novel coronavirus. *J Clin Microbiol*. 2003; **41**: 4521-4.
