# Supplementary material for: Microorganisms Causing Community-Acquired Acute Bronchitis: The Role of Bacterial Infection
Source: PLoS One. 2016 Oct 27;11(10):e0165553. doi: 10.1371/journal.pone.0165553 (PMC5082923; doi:10.1371/journal.pone.0165553)
Supplement: S1 Table — (DOCX) [file pone.0165553.s002.docx]

**SUPPLEMENTARY INFORMATION**

**Microorganisms causing community-acquired acute bronchitis: the role of bacterial infection**

Ji Young Park, Sunghoon Park, Sun Hwa Lee, Myung Goo Lee, Yong Bum Park, Kil Chan Oh, Jae-Myung Lee, Do Il Kim, Ki-Hyun Seo, Kyeong-Cheol Shin, Kwang Ha Yoo, Yongchun Ko, Seung Hun Jang, Ki-Suck Jung , and Yong Il Hwang

**S1 Table. Comparison of clinical characteristics in patients with acceptable sputum and unacceptable sputum**

| **Variables** | **Acceptable sputum**  **n=291** | **Unacceptable sputum**  **n=520** | **Total**  **n=811** | ***P* value** |
| --- | --- | --- | --- | --- |
| Mean age, yr | 49.4 ± 17.3 | 47.6 ± 16.4 | 48.2 ± 16.8 | 0.074 |
| Gender, male | 123 (42.3%) | 234 (45.0%) | 357 (44.0%) | 0.452 |
| Current smoker | 56 (19.2%) | 109 (21.0%) | 165 (20.3%) | 0.560 |
| Systemic disease |  |  |  |  |
| Hypertension | 44 (15.1%) | 83 (16.0%) | 127 (15.7%) | 0.752 |
| Diabetes mellitus | 21 (7.2%) | 28 (5.4%) | 49 (6.0%) | 0.294 |
| Chronic heart disease | 4 (1.4%) | 13 (2.5%) | 17 (2.1%) | 0.283 |
| Chronic kidney disease | 1 (0.3%) | 3 (0.6%) | 4 (0.5%) | 0.649 |
| Cerebrovascular disease | 2 (0.7%) | 3 (0.6%) | 5 (0.6%) | 0.847 |
| Pulmonary comorbidities |  |  |  |  |
| Asthma only | 29 (10.0%) | 37 (7.1%) | 66 (8.1%) | 0.155 |
| COPD only | 27 (9.3%) | 14 (2.7%) | 41 (5.1%) | <0.001 |
| Bronchiectasis only | 12 (4.1%) | 19 (3.7%) | 31 (3.8%) | 0.738 |
| Combined lung diseases | 8 (2.7%) | 6 (1.2%) | 14 (1.7%) | 0.094 |
| Symptoms |  |  |  |  |
| Duration of cough, day | 6.98 ± 5.15 | 8.67 ± 7.06 | 8.06 ± 6.49 | <0.001 |
| Sputum amount  (≥ moderate) | 156 (53.6%) | 179 (34.4%) | 335 (41.3%) | <0.001 |
| Purulent sputum | 165 (56.7%) | 224 (43.2%) | 389 (48.1%) | <0.001 |
| Rhinorrhea | 111 (38.1%) | 172 (33.1%) | 283 (34.9%) | 0.146 |
| Sore throat | 108 (37.1%) | 164 (31.5%) | 272 (33.5%) | 0.107 |
| Dyspnea | 33 (11.3%) | 56 (10.8%) | 89 (11.0%) | 0.803 |
| Fever | 16 (5.5%) | 16 (3.1%) | 32 (3.9%) | 0.089 |
| Signs |  |  |  |  |
| Wheezing | 34 (11.7%) | 30 (5.8%) | 64 (7.9%) | 0.003 |
| Crackle | 35 (12.0%) | 39 (7.5%) | 74 (9.1%) | 0.032 |
| Rhonchi | 108 (37.1%) | 132 (25.4%) | 240 (29.6%) | <0.001 |
| Stridor | 10 (3.4%) | 6 (1.2%) | 16 (2.0%) | 0.025 |

* Acceptable sputum was defined by satisfying Murray-Washington classification degree IV or V.

Abbreviations: COPD, chronic obstructive pulmonary disease. Combined lung diseases include Asthma + COPD, asthma + BE, COPD + BE, and all three diseases together.
