# Supplementary material for: Microorganisms Causing Community-Acquired Acute Bronchitis: The Role of Bacterial Infection
Source: PLoS One. 2016 Oct 27;11(10):e0165553. doi: 10.1371/journal.pone.0165553 (PMC5082923; doi:10.1371/journal.pone.0165553)
Supplement: S2 Table — (DOCX) [file pone.0165553.s003.docx]

**SUPPLEMENTARY INFORMATION**

**Microorganisms causing community-acquired acute bronchitis: the role of bacterial infection**

Ji Young Park, Sunghoon Park, Sun Hwa Lee, Myung Goo Lee, Yong Bum Park, Kil Chan Oh, Jae-Myung Lee, Do Il Kim, Ki-Hyun Seo, Kyeong-Cheol Shin, Kwang Ha Yoo, Yongchun Ko, Seung Hun Jang, Ki-Suck Jung , and Yong Il Hwang

**S2 Table. The distribution of mixed infections in patients with acceptable sputum**

| **Pathogens** | **Subjects (n)** |
| --- | --- |
| **Mixed infection with virus and bacteria** |  |
| Rhinovirus plus: |  |
| *H. influenza* | 8 |
| *H. influenzae and S. pneumoniae and B. pertussis* | 1 |
| *H. influenzae and S. pneumonia and M. pneumonia* | 1 |
| *H. influenzae and K. pneumoniae* | 1 |
| *H. influenzae and P. aeruginosa* | 1 |
| *H. influenzae and enterovirus* | 1 |
| *K. pneumonia* | 4 |
| *K. pneumonia and B. pertussis* | 1 |
| *K. pneumonia and Coronavirus* | 1 |
| *S. pneumonia* | 3 |
| *S. pneumonia and M. catarrhalis* | 1 |
| *S. pneumonia and enterovirus* | 1 |
| *M. catarrhalis* | 1 |
| *M. catarrhalis and L. pneumophila* | 2 |
| *M. catarrhalis and L. pneumophila and adenovirus* | 1 |
| *S. aureus* | 2 |
| *S. aureus and adenovirus* | 1 |
| *S. aureus and P. aeruginosa* | 1 |
| *P. aeruginosa* | 2 |
| *B. pertussis* | 1 |
| Adenovirus plus: |  |
| *H. influenza* | 1 |
| *H. influenza and coronavirus* | 1 |
| Coronavirus plus: |  |
| *K. pneumonia* | 1 |
| *M. pneumonia* | 1 |
| *B. pertussis* | 1 |
| Parainfluenza virus plus: |  |
| *K. pneumonia* | 1 |
| *H. influenzae and S. pneumoniae* | 1 |
| *H. influenzae and S. pneumonia and K. pneumoniae* | 1 |
| RSV plus: |  |
| *H. influenza* | 1 |
| *M. catarrhalis* | 1 |
| *M. pneumonia* | 1 |
| Human metapneumovirus |  |
| *K. pneumonia* | 1 |
| *S. pneumonia and S. aureus* | 1 |
| Influenza A plus: |  |
| *S. pneumonia* | 1 |
| Influenza B plus: |  |
| *S. pneumoniae* | 1 |
| **Co-infection with multiple bacteria, without virus** |  |
| *H. influenzae* plus: |  |
| *S. pneumoniae* | 2 |
| *S. pneumonia and K. pneumoniae* | 1 |
| *M. catarrhalis* | 1 |
| *S. aureus* | 1 |
| *S. pneumonia* plus: |  |
| *S. aureus* | 1 |
| *P. aeruginosa* | 1 |
| *M. catarrhalis* plus: |  |
| *L. pneumophila* | 5 |
| *P. aeruginosa* plus: |  |
| *L. pneumophila* | 2 |
| *M. pneumonia* plus *L. pneumophila* | 1 |
| *S. aureus and B. pertussis* | 1 |
| **Co-infection with multiple virus, without bacteria** |  |
| Rhinovirus plus: |  |
| Enterovirus | 4 |
| Adenovirus | 2 |
| Coronavirus | 3 |
| Adenovirus plus: |  |
| Coronavirus | 1 |
